# Amyloid-Related Memory Decline in Preclinical Alzheimer’s Disease Is Dependent on *APOE* ε4 and Is Detectable over 18-Months

**DOI:** 10.1371/journal.pone.0139082

**Published:** 2015-10-02

**Authors:** Christine Thai, Yen Ying Lim, Victor L. Villemagne, Simon M. Laws, David Ames, Kathryn A. Ellis, Stephanie R. Rainey-Smith, Ralph N. Martins, Colin L. Masters, Christopher C. Rowe, Paul Maruff, Australian Imaging, Biomarkers and Lifestyle (AIBL) Research Group

**Affiliations:** 1 Department of Psychology, Royal Melbourne Institute of Technology, Melbourne, Australia; 2 The Florey Institute of Neuroscience and Mental Health, Parkville, Victoria, Australia; 3 Department of Neurology, Warren Alpert School of Medicine, Brown University, Providence, Rhode Island, United States of America; 4 Department of Neurology, Rhode Island Hospital, Providence, Rhode Island, United States of America; 5 Department of Nuclear Medicine and Centre for PET, Austin Health, Heidelberg, Victoria, Australia; 6 Department of Medicine, Austin Health, The University of Melbourne, Heidelberg, Victoria, Australia; 7 Centre of Excellence for Alzheimer’s Disease Research and Care, Edith Cowan University, Joondalup, Western Australia, Australia; 8 Sir James McCusker Alzheimer’s Disease Research Unit, Hollywood Private Hospital, Perth, Western Australia, Australia; 9 Co-operative Research Centre for Mental Health, Perth, Western Australia, Australia; 10 Academic Unit for Psychiatry of Old Age, St. Vincent’s Health, The University of Melbourne, Kew, Victoria, Australia; 11 National Ageing Research Institute, Parkville, Victoria, Australia; 12 Cogstate Ltd., Melbourne, Victoria, Australia; Duke-NUS Graduate Medical School, SINGAPORE

## Abstract

High levels of β-amyloid (Aβ) in the brain and carriage of the *APOE* ε4 allele have each been linked to cognitive impairment in cognitively normal (CN) older adults. However, the relationship between these two biomarkers and cognitive decline is unclear. The aim of this study was to investigate the relationship between cerebral Aβ level, *APOE* ε4 carrier status, and cognitive decline over 18 months, in 317 cognitively healthy (CN) older adults (47.6% males, 52.4% females) aged between 60 and 89 years (Mean = 69.9, SD = 6.8). Cognition was assessed using the Cogstate Brief Battery (CBB) and the California Verbal Learning Test, Second Edition (CVLT-II). Planned comparisons indicated that CN older adults with high Aβ who were also APOE ε4 carriers demonstrated the most pronounced decline in learning and working memory. In CN older adults who were *APOE* ε4 non-carriers, high Aβ was unrelated to cognitive decline in learning and working memory. Carriage of *APOE* ε4 in CN older adults with low Aβ was associated with a significantly increased rate of decline in learning and unexpectedly, improved cognitive performance on measures of verbal episodic memory over 18 months. These results suggest that Aβ and *APOE* ε4 interact to increase the rate of cognitive decline in CN older adults and provide further support for the use of Aβ and *APOE* ε4 as biomarkers of early Alzheimer’s disease.

## Introduction

In cognitively normal (CN) older adults, both an abnormal level of amyloid-β (Aβ+) and carriage of the apolipoprotein E (*APOE*) ε4 allele have been identified as risk factors for cognitive decline and the development of Alzheimer’s disease (AD) [1–4]. The amyloid cascade model of AD posits that Aβ accumulation gives rise to neurodegeneration, loss of neurotransmitter function, and ultimately, cognitive impairment and dementia [5,6]. While *APOE* ε4 influences cell death through promoting the toxic effects of Aβ, it also contributes to AD pathogenesis indirectly by reducing synaptic plasticity and increasing risk for cerebrovascular events and/or mitochondrial dysfunction [7–11]. *In vitro* studies also indicate that the effects of Aβ and *APOE* ε4 can interact to promote cell death [12–14]. The results of recent clinical studies in humans also support the hypothesis that *APOE* ε4 and Aβ interact to influence AD disease progression. Specifically, while the deleterious effects of Aβ on cognitive function, particularly in episodic memory, in CN adults was well known [15–17], the magnitude of Aβ related decline in episodic memory over 36-54 months in Aβ+ CN adults was increased further in individuals who carried the *APOE* ε4 allele [18–20]. However, interactions between ε4 and Aβ related cognitive decline in CN adults have not been observed when studies have been restricted to periods of 18 months [15,16]. Taken together, these data suggest that while ε4 does exacerbate Aβ related cognitive decline in CNs, this interaction may only become evident over longer periods of assessment.

Understanding how *APOE* ε4 moderates Aβ toxicity in CN adults provides an exciting opportunity for the development of anti-Aβ therapies based on the protective actions of the apoE2 and apoE3 isoforms [10,21]. If this were the case, such therapies could be described as alleviating, or even curing, the effects of *APOE* ε4. Given the accepted importance of this lipoprotein in epidemiological and experimental models of AD [21,22], such statements would most likely be accepted as being reflective of effects that are clinically important. Furthermore, as clinical trials of drugs designed to reduce Aβ toxicity are typically conducted over intervals of 18-months [23,24], it is important to determine whether the ε4 exacerbation of Aβ related cognitive decline requires more than 18 months to become evident, or whether the absence of such effects in studies of this duration was related to the relatively small sample sizes studied did not provide sufficient statistical power to detect the interaction of apoe4 and amyloid on memory.

The aim of the current study was to characterize *APOE* ε4-related acceleration of Aβ-related cognitive decline over an 18-month test-retest interval on a computerized cognitive battery in a large group of CN older adults whose ε4 and Aβ status was known. The hypothesis was that in CN older adults, cognitive decline over 18 months would be greatest in those who were Aβ+ and ε4 carriers.

## Materials and Methods

CN older adults (n=317) enrolled in the Australian Imaging, Biomarkers and Lifestyle (AIBL) study who had undergone positron emission tomography (PET) neuroimaging for Aβ and who had completed cognitive assessments at a baseline and 18 month assessment participated in this study. The process of recruitment and diagnostic classification of CN older adults has been described previously [25]. Participants were excluded from the study if they had any of the following: schizophrenia, depression (15-item Geriatric Depression Scale [GDS] score ≥ 6), Parkinson’s disease, cancer (other than basal cell skin carcinoma) within the last two years, obstructive sleep apnoea, symptomatic stroke, uncontrolled diabetes, or current regular alcohol use exceeding two standard drinks per day for women or four per day for men. Demographic data including age, sex, education level was collected and scores for premorbid IQ, and depressive and anxiety symptoms (using the GDS and the Hospital Anxiety and Depression Scale) were also collected at screening (see Table 1). The study was approved by and complied with the regulations of the institutional research and ethics committees of Austin Health, St. Vincent’s Health, Hollywood Private Hospital and Edith Cowan University [25]. All participants provided written informed consent prior to being tested.

**Table 1:** Demographic and clinical characteristics.

	Overall	Aβ- ε4 non-carriers	Aβ- ε4 carriers	Aβ+ ε4 non-carriers	Aβ+ ε4 carriers
*N*	317	182	59	31	45
N (%) female	166 (52.4%)	93 (51.1%)	32 (54.2%)	17 (54.8%)	24 (53.3%)
N (%) high Aβ +	40 (12.6%)	n/a	n/a	14 (45.2%)	26 (57.8%)
Age, mean (SD)	69.9 (6.8)	68.8 (6.0)	66.5 (4.9)	76.0 (7.2)	72.3 (7.1)
Education (years)	13-15	13-15	15+	13-15	13-15
Premorbid IQ, mean (SD)	108.58 (7.02)	108.46 (6.87)	107.36 (7.83)	111.28 (6.22)	108.89 (6.34)
GDS*, mean (SD)	0.84 (1.31)	0.90 (1.39)	1.02 (1.51)	0.48 (0.87)	0.62 (0.89)
HADS-D^@^, mean (SD)	2.61 (2.51)	2.59 (2.31)	2.73 (2.01)	1.83 (1.49)	2.98 (2.76)
HADS-A^#^, mean (SD)	4.20 (2.80)	4.24 (2.75)	4.02 (2.84)	3.07 (2.00)	5.13 (3.29)
CDR-SB^$^, mean (SD)	0.43 (0.17)	0.04 (0.15)	0.05 (0.14)	0.07 (0.18)	0.02 (0.10)
MMSE^^^, mean (SD)	28.87 (1.19)	28.90 (1.20)	28.82 (1.24)	28.79 (1.24)	28.71 (1.16)

*GDS = Geriatric Depression Scale

^@^HADS-D = Hospital Anxiety and Depression Scale, Depression Subscale

^#^HADS-A = Hospital Anxiety and Depression Scale, Anxiety Subscale

^$^CDR-SB = Clinical Dementia Rating scale, Sum of Boxes score

^^^MMSE = Mini Mental State Examination

Aβ PET imaging was conducted using either ^11^C-Pittsburg-compound B (^11^C-PiB), ^18^F-florbetapir, or ^18^F-flutemetamol. A 30-minute acquisition was started 40-minutes post-injection of PiB, a 20-minute acquisition was performed 50-minutes post-injection of florbetapir and 90-minutes post-injection of flutemetamol. For PiB-PET, standardized uptake value (SUV) data were summed and normalized to the cerebellar cortex SUV, resulting in a region-to-cerebellar ratio termed SUV ratio (SUVR). The whole cerebellum was the reference region for florbetapir, while for flutemetamol, the reference region was the pons. High Aβ was classified as SUVR≥ 1.5 for ^11^C-PiB, SUVR≥1.1 for ^18^F-florbetapir, and SUVR≥0.62 for ^18^F-flutemetamol. Aβ+ levels were further classified as being “high” Aβ+ (SUVR PiB>1.9; flutemetamol>0.82; florbetapir>1.29) or “low” Aβ+ (SUVR PiB=1.5-1.9; flutemetamol=0.62-0.82; florbetapir=1.11-1.29) [26,27]. 80ml fasted blood samples were collected from each participant, of which 0.5 ml was sent to a clinical pathology laboratory for *APOE* genotyping.

Cognition was assessed using the CVLT-II (immediate and delayed recall scores) and the Cogstate Brief Battery (CBB) because these measures have demonstrated sensitivity to cognitive impairment in mild cognitive impairment (MCI) and AD and to cognitive decline associated with Aβ+ in preclinical AD [15,28]. The CVLT-II involves participants learning a 16-item word list over five trials. The CBB consists of four computerised card games that measure cognitive performance across four domains: psychomotor function, visual attention, working memory, and learning. The Detection task (DET) assesses psychomotor function. During the task, a card is presented face down in the centre of the computer screen and when the card turns face up, participants are required to press the yes button as quickly as possible. Psychomotor function was assessed by measuring the time (milliseconds) taken to respond correctly, which was normalized using a logarithmic base 10 (log_10_) transformation. The Identification task (IDN) assesses visual attention. During the task, participants respond to the question “is the card red?” using yes and no buttons. Visual attention was assessed by measuring the time (milliseconds) taken to respond correctly, which was normalized using a log_10_ transformation. The One Card Learning task (OCL) assesses learning. During the task, participants use yes and no buttons to respond to the question “have you seen this card before in this task?” as cards are presented. During the task, six playing cards are repeatedly displayed amongst distractors (non-repeating cards). Learning was assessed by measuring the accuracy of participant response, which was quantified as the proportion of correct trials during the task, and normalized using an arcsine square-root transformation. The One Back task (OBK) assesses working memory. During the task, participants use yes and no buttons to respond to the question “is the previous card the same?” Working memory was assessed by measuring the accuracy of participant responses, which was quantified as the proportion of correct trials during the task, and normalized using an arcsine square-root transformation. Two composite scores were also constructed from these measures, a Psychomotor/Attention (DETIDN) composite, and a Learning/Working Memory (OCLOBK) composite. Data for ratings on the Mini Mental State Examination (MMSE) and the Clinical Dementia Rating (CDR) scale were also collected for the baseline and 18 month assessments.

### Data Analysis

Data for each cognitive outcome measure was analysed using a series of pairwise comparisons (*df* =3) conducted within an analysis of covariance (ANCOVA). These comparisons were set to reflect the hypotheses that compared to Aβ- ε4 non-carriers, cognitive decline would be greatest in Aβ+ ε4 carriers. In each ANCOVA, Aβ and *APOE* carrier status were entered as fixed factors, and age, premorbid IQ, levels of anxiety symptoms, and the baseline measure of the task were entered as covariates (as these were found to differ between groups Table 1). The 18-month scores for each measure were entered as the dependent variable. The magnitude of difference for change in cognitive performance over 18 months from the Aβ- ε4 non-carrier group was calculated for each group using the estimated marginal means at 18 months and differences between groups were expressed using Cohen’s *d* and 95% confidence intervals (95% CIs).

## Results

### Demographic and clinical characteristics

Demographic and clinical characteristics for the overall sample and each Aβ*/APOE* group are shown in Table 1. Differences between groups were identified for age [F(3,313) = 17.81, *p*<.001], premorbid IQ [F(3,312) = 2.96. *p*=.03] and levels of anxiety symptoms [F(3,311) = 3.05, *p*=.03]. Groups did not differ on any other clinical or demographic characteristic (all *p*’s >.05). Consequently, age, premorbid IQ and levels of anxiety symptoms were included as covariates in comparisons of cognitive performance between groups. There were no differences in the proportion of ε4 carriers or non-carriers who were classified as high Aβ+, χ^2^ =1.17, *p*=.28 (Table 1).

### Effect of Aβ and *APOE* ε4 on cognitive decline over 18 months

**Fig 1:**
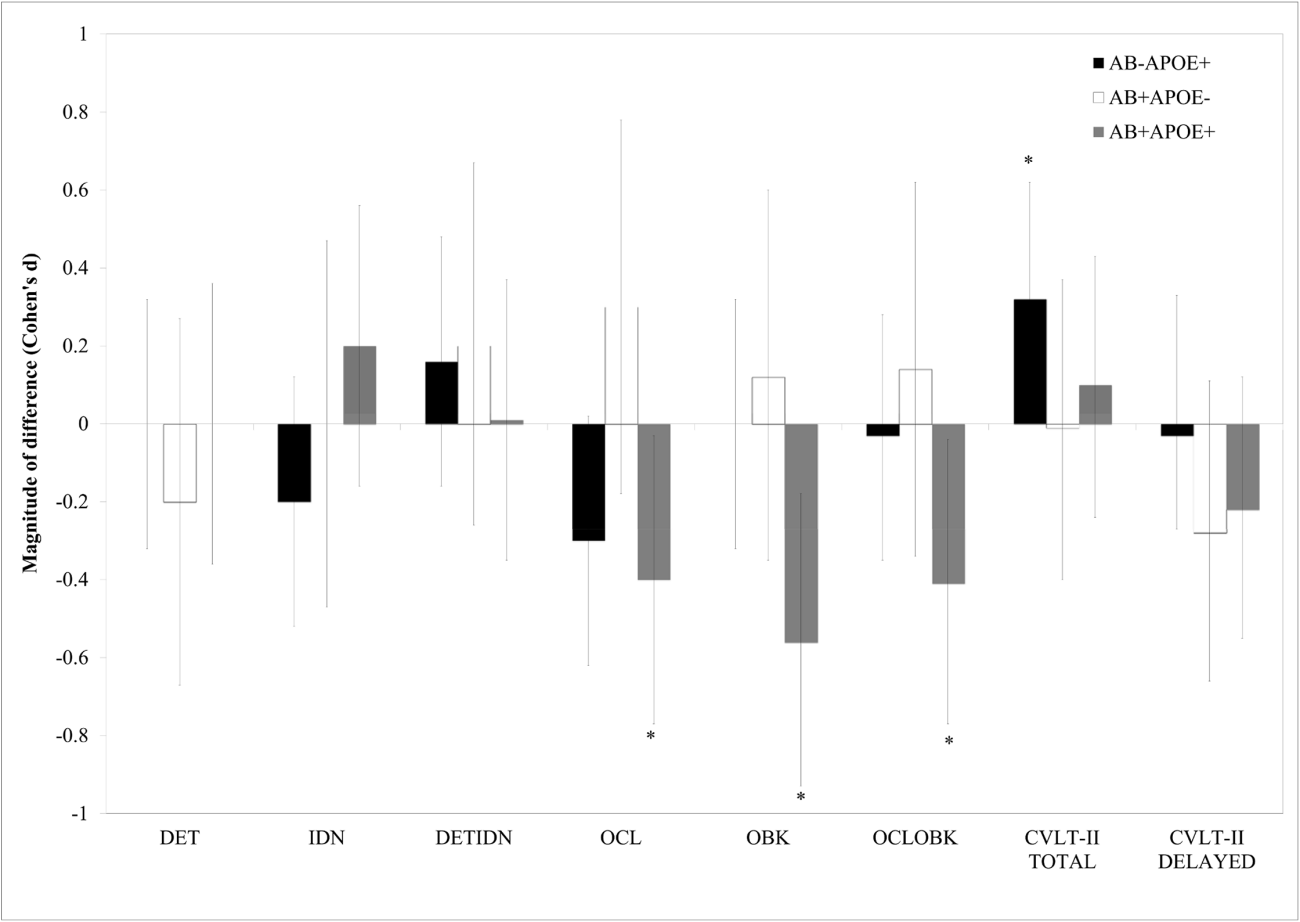
Magnitude of difference for cognitive decline over 18 months, relative to the Aβ- ε4 non-carrier group. 0 line represents the Aβ- ε4 non-carrier group; error bars represent 95% confidence intervals. *p<.05.

Baseline- and covariate-adjusted means for the 18-month assessment are shown in Table 2, and the magnitude of difference between groups is shown in Fig 1. In the Aβ- groups, no differences between ε4 carriers and non-carriers were observed for any of the cognitive measures. Compared to Aβ- ε4 non-carriers, Aβ- ε4 carriers showed a slight improvement on the CVLT-II immediate total recall at 18 months, and no decline on any other cognitive measure. In contrast, compared to Aβ- ε4 non-carriers, Aβ+ ε4 carriers performed significantly worse at 18 months on the OCL, OBK and the OCLOBK composite scores, with each of these differences between groups moderate in magnitude (Table 2).

**Table 2:** Baseline and covariate adjusted mean scores at 18 months for each group and Cohen’s d of the difference in mean score change over 18 months, relative to the Aβ- ε4 non-carrier group. Abbreviations: DETIDN, Psychomotor/Attention composite; DET, Detection task; IDN, Identification task; OCLOBK, Learning/Working Memory composite; OCL, One Card Learning task; OBK, One Back Learning task; CVLT-II Total, California Verbal Learning Test, Second Edition, total recall; CVLT-II Delay, CVLT-II, 20 minute delayed recall. NOTE. Bolded values are significant at the *p* < .05 level. Means are adjusted for age, premorbid IQ, levels of anxiety symptoms, and baseline performance.

	18-month adjusted Mean (SD)				Cohen’s *d* (95% CI) (vs. Aβ- ε4 non-carriers)		
Task	Aβ- ε4 non-carriers	Aβ- ε4 carriers	Aβ+ ε4 non-carriers	Aβ+ ε4 carriers	Aβ- ε4 carriers	Aβ+ ε4 non-carriers	Aβ+ ε4 carriers
DETIDN	-0.03 (0.68)	0.08 (0.70)	0.11 (0.71)	-0.02 (0.70)	0.16 [-0.16, 0.48]	0.20 [-0.26, 0.67]	0.01 [-0.35, 0.37]
DET	2.52 (0.10)	2.52 (0.10)	2.50 (0.10)	2.52 (0.10)	0.00 [-0.52, 0.12]	-0.20 [-0.67, 0.27]	0.00 [-0.36, 0.36]
IDN	2.71 (0.05)	2.70 (0.05)	2.71 (0.05)	2.72 (0.05)	-0.20 [-0.52, 0.12]	0.00 [-0.47, 0.47]	0.20 [-0.16, 0.56]
OCLOBK	0.07 (0.93)	0.04 (0.94)	0.20 (0.96)	-0.31 (0.95)	-0.03 [-0.35, 0.28]	0.14 [-0.34, 0.62]	**-0.41 [-0.77, -0.04]**
OCL	1.03 (0.10)	1.00 (0.10)	1.06 (0.10)	0.99 (0.10)	-0.30 [-0.62, 0.02]	0.30 [-0.18, 0.78]	**-0.40 [-0.77, -0.03]**
OBK	1.34 (0.16)	1.34 (0.16)	1.36 (0.17)	1.25 (0.17)	0.00 [-0.32, 0.32]	0.12 [-0.35, 0.60]	**-0.56 [-0.93, -0.18]**
CVLT-II Total	49.58 (7.54)	52.01 (7.68)	49.47 (7.94)	50.31 (7.64)	**0.32 [0.02, 0.62]**	-0.01 [-0.40, 0.37]	0.10 [-0.24, 0.43]
CVLT-II Delay	11.83 (2.57)	11.91 (2.62)	11.11 (2.71)	11.27 (2.61)	0.03 [-0.27, 0.33]	-0.28 [-0.66, 0.11]	-0.22 [-0.55, 0.12]

## Discussion

The results of this study support the hypothesis that in CN older adults, cognitive decline over 18 months is greatest in Aβ+ CN older adults who were ε4 carriers. In fact, the only statistically significant cognitive decline identified in the current study was between Aβ+ ε4 carriers and Aβ- ε4 non-carriers (Table 2). This group showed a decline over 18 months, that was moderate in magnitude, on measures of visual learning (Cogstate OCL task), working memory (Cogstate OBK Task) and a learning/working memory composite score (OCLOBK; Table 2). Aβ+ ε4 carriers also showed decline on the measure of verbal delayed recall but the magnitude of this was not sufficient to reach statistical significance (Table 2). In contrast, both Aβ+ ε4 non-carriers and Aβ- ε4 carriers showed no decline over the 18 months for any aspect of cognitive function measured (Table 2). Interestingly, Aβ- ε4 carriers showed a decline of a moderate magnitude over 18 months, but only for the measure of visual learning and this decline was not sufficient to reach statistical significance. Considered together, these data in general suggest that in CN older adults, memory decline over relatively short intervals requires the additive effects of Aβ+ and *APOE* ε4. These results are largely consistent with recent studies conducted over 36-54 months which demonstrated that Aβ related decline in memory was much greater if individuals also were *APOE* ε4 carriers [18,19].

The findings of the current study therefore suggest that acceleration of Aβ+ related memory decline is evident in carriers of the *APOE* ε4 allele and is sufficiently large enough to be evident over relatively short time periods. As such, it would be prudent for clinical trials of putative disease modifying drugs to stratify their Aβ+ samples according to *APOE* ε4 status when memory measures are included as the endpoint. The data from this study can also be used as a foundation for computation of statistical power calculations that may be relevant to the design of clinical trials for early AD. For example, using the data presented in Table 2, the extent to which ε4 increased Aβ related memory decline is moderate in magnitude (i.e., Aβ+ ε4 non-carriers vs. Aβ+ ε4 carriers *d* = 0.53). As such, the sample size required to detect a therapeutic effect of alleviating the effects of ε4 would be 57 per group (assuming *d* = 0.53, α = 0.05, power = 0.80, two-tailed distribution, and equal sample size in both groups).

The absence of any cognitive decline in Aβ+ ε4 non-carriers in the current study suggests that, by itself Aβ+ was not sufficient for the manifestation of cognitive decline in CN older adults. Similarly, when CN older adults were followed over much longer periods (e.g., 4.5 years), Aβ+ ε4 non-carriers also showed very little decline in memory or other aspects of cognitive function [18,19]. These data are now becoming strong enough to suggest a revision for the current pathological models of AD which propose that in the preclinical stage of the disease, Aβ+ by itself is the necessary condition for cognitive decline [29,30]. However, few studies have stratified their Aβ+ CN older groups according to *APOE* ε4 status because the number of CN ε4 carriers present in preclinical AD samples has been relatively small and therefore studies have been unable to directly test these pathological models of AD. Given the current findings and their consistency with previous data gathered over longer time intervals it is possible that the Aβ related cognitive decline reported in CN older adults is being driven also by the additive effects of the *APOE* ε4 allele [18,19]. The current findings also suggest that current models of AD need to consider the effect of genetic polymorphisms in moderating the rate by which neuropathological processes occur.

The current study found that, in the absence of Aβ+, *APOE* ε4 also had no effect on cognitive function. The two previous studies that examined the extent to which Aβ+ and *APOE* ε4 influenced cognitive decline also observed no effects of *APOE* ε4 carriage in Aβ- CN adults [18,19]. While a series of well-designed and large prospective studies have shown that *APOE* ε4 carriage is associated with increased decline in episodic memory and executive function [31–33], none of these have measured Aβ levels in their sample. Given that ε4 carriage increases the risk of Aβ+ it is likely in these studies that the effect of *APOE* ε4 on cognitive decline is mainly due to the interaction between Aβ and ε4 rather than an effect of ε4 by itself [34,35]. Caselli and colleagues have emphasised that within groups of cognitively normal ε4 carriers, those who are homozygous for the ε4 allele show much greater decline than heterozygotes [31]. This raises a challenge for interpreting the current data, as well as for data from previous studies reporting interactions between Aβ and *APOE* ε4, as none of these studies stratified their *APOE* ε4 groups according to zygosity. While it is possible that Aβ related cognitive decline would be greater in *APOE* ε4 homozygotes compared to *APOE* ε4 heterozygotes, a test of this hypothesis would require the recruitment of very large samples of Aβ+ CN older adults and may therefore be better investigated using meta-analytic methods.

Studies in humans and transgenic mice have shown that *APOE* is involved in AD pathogenesis both directly, through increasing Aβ accumulation, reducing clearance of Aβ, or modifying Aβ-synaptic toxicity; and indirectly, through reducing synaptic plasticity, increasing neuroinflammation or affecting the concurrence of cerebrovascular events [10]. Similarly, one study has demonstrated that in humans, ε4, independent of Aβ, is associated with brain hypometabolism [36]. However, as the AIBL study excluded individuals with uncontrolled cardiovascular disease, the risk of concomitant cerebrovascular events over the 18-month interval is very low. Therefore it is possible that the faster memory decline observed in healthy Aβ+ ɛ4 carriers was due to the direct effects of *APOE* on Aβ accumulation.

There is now much evidence from *in vitro*, animal, and human studies showing that isoforms of the apoE protein also affect risk for AD by differentially modulating Aβ clearance and accumulation in the brain; apoE4 increases Aβ accumulation and disrupts clearance relative to other apoE isoforms [11–14]. A recent *in vivo* study showed that the clearance of soluble Aβ in the brain interstitial fluid (ISF) depends on the isoform of human apoE expressed (apoE4 < apoE3 ≤ apoE2) [14]. This hypothesis accords with Aβ mouse models, which report that disruption of the apoE-Aβ interaction can result in a significant reduction of Aβ plaques. Taken together, these data suggest that Aβ+ ɛ4 related cognitive decline in preclinical AD may make an ideal target for drugs that moderate neurodegeneration arising from Aβ+, or from the interaction between Aβ+ and ɛ4 [10,21].

When considering the results of this study, as has been described previously, an important caveat is that the AIBL study is not a population-based sample [19, 29]. Cognitively normal older adults in the AIBL study were highly educated, and few had existing or untreated medical, neurological or psychiatric illnesses. Secondly, an extensive investigation of cognitive function was not conducted. The tasks used here were chosen based on their brevity, test-retest reliability, demonstrated sensitivity to short-term Aβ-related cognitive decline, and their use in current large secondary prevention clinical trials in preclinical AD. An exploration of more detailed neuropsychological tests over the same short-term period will be useful in further elucidating the nature of Aβ and ε4 related decline in cognitive function. Finally, as three radioligands were used to measure Aβ, SUVR data could not be integrated to form a single continuous measure of Aβ burden. However, there was no relationship between the proportion of individuals who were high and low Aβ+ in Aβ+ ε4 carriers and Aβ+ ε4 non-carriers, suggesting that the faster decline observed in the Aβ+ ε4 carriers was not due to more advanced disease at enrolment. However, these caveats notwithstanding, the results of the current study suggest that *APOE* ε4 exacerbates the rate of Aβ-related memory decline in CN older adults, and support the consideration of *APOE* ε4 status when interpreting cognitive endpoints of clinical trials in AD. Further, the current results support the growing number of studies reporting the synergistic effect of *APOE* and Aβ in affecting cognitive decline in preclinical AD. As the rate of cognitive decline in preclinical AD is significantly increased by the presence of *APOE* ε4, with some reports showing that Aβ+ ε4 non-carriers do not show any decline in cognitive function even over 4-5 years, it is imperative that current secondary prevention clinical trials in preclinical AD consider the ε4 status of individuals enrolled.
